# Individual Circadian Preference, Shift Work, and Risk of Medication Errors: A Cross-Sectional Web Survey among Italian Midwives

**DOI:** 10.3390/ijerph17165810

**Published:** 2020-08-11

**Authors:** Rosaria Cappadona, Emanuele Di Simone, Alfredo De Giorgi, Benedetta Boari, Marco Di Muzio, Pantaleo Greco, Roberto Manfredini, María Aurora Rodríguez-Borrego, Fabio Fabbian, Pablo Jesús López-Soto

**Affiliations:** 1 Department of Medical Sciences, University of Ferrara, 44121 Ferrara, Italy; rosaria.cappadona@unife.it (R.C.); pantaleo.greco@unife.it (P.G.); roberto.manfredini@unife.it (R.M.); 2 Obstetrics and Gynecology Unit, Azienda Ospedaliero-Universitaria S. Anna, 44121 Ferrara, Italy; 3 Department of Nursing, Instituto Maimónides de Investigación Biomédica de Córdoba (IMIBIC), 14071 Córdoba, Spain; en1robom@uco.es (M.A.R.-B.); n82losop@uco.es (P.J.L.-S.); 4 Department of Clinical and Molecular Medicine, Sapienza University of Rome, 00185 Rome, Italy; emanuele.disimone@uniroma1.it (E.D.S.); marco.dimuzio@uniroma1.it (M.D.M.); 5 Clinica Medica Unit, Azienda Ospedaliero-Universitaria S. Anna, 44121 Ferrara, Italy; degiorgialfredo@libero.it (A.D.G.); benedetta.boari@unife.it (B.B.); 6 Department of Nursing Pharmacology and Physiotherapy, University of Córdoba, 14071 Córdoba, Spain

**Keywords:** circadian rhythm, chronotype, midwives, Morningness–Eveningness Questionnaire (MEQ), near misses, nurses, rhythms desynchronization, risk of medication errors, shift work, sleep

## Abstract

Background: In order to explore the possible association between chronotype and risk of medication errors and chronotype in Italian midwives, we conducted a web-based survey. The questionnaire comprised three main components: (1) demographic information, previous working experience, actual working schedule; (2) individual chronotype, either calculated by Morningness–Eveningness Questionnaire (MEQ); (3) self-perception of risk of medication error. Results: Midwives (*n* = 401) responded “yes, at least once” to the question dealing with self-perception of risk of medication error in 48.1% of cases. Cluster analysis showed that perception of risk of medication errors was associated with class of age 31–35 years, shift work schedule, working experience 6–10 years, and Intermediate-type MEQ score. Conclusions: Perception of the risk of medication errors is present in near one out of two midwives in Italy. In particular, younger midwives with lower working experience, engaged in shift work, and belonging to an Intermediate chronotype, seem to be at higher risk of potential medication error. Since early morning hours seem to represent highest risk frame for female healthcare workers, shift work is not always aligned with individual circadian preference. Assessment of chronotype could represent a method to identify healthcare personnel at higher risk of circadian disruption.

## 1. Introduction

Chronobiology is a biomedical discipline devoted to the study of biological rhythms. Biological rhythms exist at any level of living organisms and, according to their cycle length, are classified into: (a) circadian rhythms (from the Latin circa-dies, characterized by a period of ~24 h), (b) ultradian rhythms (period <24 h), and c) infradian rhythms (period >24 h) [1]. Circadian rhythms are the most commonly and widely studied biological rhythms, but they are not strictly the same in all persons, since an individual circadian preference (the so-called chronotype) closely linked to biological and psychological variables, exists. Horne & Ostberg first spoke of possible individual differences in circadian attitudes, the so-called chronotype [2]. By means of a simple a self-assessment Morningness–Eveningness Questionnaire (MEQ), they identified Morning-types (M-type, more active early in the day), Evening-types (E-type, more active later in the day), and neither type or Intermediate (I-type). As for chronotype distribution, data based on human population in the temperate region seem to show a Gaussian curve, with 10% M-type, 10% E-type, and 80% I-type [3]. Moreover, differences by sex and age exist as well, with men on average more evening-oriented than women, although these differences reduce with time [3]. In fact, young women are more morning-oriented than young men, but older women are less morning-oriented than older men [4].

*Chronotype.* A growing body of research indicates that evening chronotype may be associated with a series of unfavourable conditions. A review from our group analysed the available literature to evaluate the relationships between chronotype, gender, and different aspects of health, such as general health and metabolism, psychological health, and sleep and sleep-related problems (Table 1) [5,6]. As a general rule, M-types cope better with the synchrony effect than E-types and are able to adapt to unfavourable circumstances. At suboptimal times, M-types solved the analogy detection task faster, with the same accuracy and without the investment of more cognitive resources. They also showed greater alertness and wakefulness. At optimal times of day, M-types had more cognitive resources available to allocate in the case of more demanding conditions. E-types appear less able to adapt to suboptimal times, because they have to deal with social jetlag and decreased self-control [7]. In fact, a recent systematic review reported that female nurses with an evening-oriented preference suffer more problems of insomnia, sleepiness, fatigue, and anxiety [8].

*Shift work.* Circadian rhythms are entrained by the light/dark alternation and a series of desynchronizing factors, such as exposure to light at night, jetlag, shift work, and daylight saving time, play a crucial role in disrupting the individual organization of circadian rhythms. Among these desynchronizing factors, shift work certainly plays crucial role, due to the wide dissemination of social request for activities warranting 24/hours/seven days a week assistance [9]. According to data from the Italian Institute of Statistics (ISTAT) survey “Working time organization: the role of atypical work schedules”, shift working involves about one worker of five in Italy (20.4%). By disaggregating according to industry, social services (which includes education, health and social services, public administration) show the highest figures (26.8%), followed by manufacturing (21.9%) and trade services, including commerce, transport, and communications (21.7%). More men than women work with shifts (respectively 21.7% and 18.4%, respectively), due mainly to shift schemes which imply night work [10]. Different disorders have been associated with shift work, with stress and sleep disorders as the most frequent ones. For example, when comparing nurses and firefighters with day workers, sleep disturbances were more frequent in shift workers than day workers [11]. Moreover, different cardio-metabolic indices, including higher waist circumference, body mass index, fasting glucose, blood pressure, and cardio-metabolic risk score have been described in night workers [12], who also showed almost three times higher association with abdominal obesity independent of age and gender than day shift workers [13].

*Errors.* Medications errors (MEs) represent a major concern of healthcare systems worldwide, and near misses represent the most reported incidents (69.3%) [14]. According to the World Health Organization, near miss is defined as “an error that has the potential to cause an adverse event (patient harm) but fails to do so because it is intercepted” [15]. Although inadequate staffing levels, workload, and working in haste have been called the most frequent causes of increased risk for omissions and other types of error and for patient harm [16], circadian misalignment, in addition to a series of health problems in shift workers secondary to the sleep deprivation, e.g., daytime sleepiness, i.e., the difficulty maintaining wakefulness and alertness during normal waking hours [17,18,19,20], can represent a crucial favouring factor for lack of performance and any kind of errors. Attentional networks are sensitive to sleep deprivation and increased time awake, and sleep duration variability appeared to moderate the association between sleep duration with overall reaction time and alerting scores [21]. Nurses’ sleep quality, immediately prior to a working 12-h shift, was shown to be more predictive of error than sleep quantity [22] and functional magnetic resonance imaging (fMRI) studies showed that task performance in nonoptimal times of the day may result in cognitive impairments leading to increased error rates and slower reaction times [23]. Moreover, sleep deprivation represents a further source of risk, not limited to the short term. In hospital shift workers, being screened positive for a sleep disorder was associated with 83% increased incidence of adverse safety outcomes in the following six months, such as motor vehicle crashes, near-miss crashes, occupational exposures, and medical errors [24].

*Health professionals.* Recent data from Canada, with reference to the year 2011, show that approximately 1.8 million Canadians (12% of the working population), were exposed to night shift work, and 45% were female. By occupation, professional occupations in health ranked second place (35% of workers), following occupations in protective services (37%) [25]. However, despite these numbers, and even if nurses and midwives make up almost 50% of the global healthcare shift working workforce, much of the research addressed to the shift work area used men [26]. Female nurses working in rotating night shift were found to have significantly lower mean scores in job satisfaction, sleep, and psychological well-being as compared to day shift workers [27] and even impaired sexual self-efficacy and sexual quality of life [28]. There is an extreme paucity of studies conducted on midwives, although they play an important role in medical care: a systematic review of sleep-related/fatigue-management including more than 8600 participants, 89% females, did not find studies conducted in midwives [29]. In the same year, a survey study by the American College of Nurse-Midwives Sleep and Safety Taskforce, conducted on more than 4350 certified nurse-midwives and midwives to identify sleepiness, found that midwives working shifts >12 h had higher rates of excessive daytime sleepiness compared with those who worked shifts of ≤12 h [30].

Based on these premises, the aim of this study was to evaluate the possible association between chronotype, shift work, and risk of medication errors in midwives, inviting a representative sample of these health care professionals by means of a web survey. Social media, in fact, has increased the popularity since it represents a convenient method for communicating on the Web, for recruiting participants for health research, and for conducting survey studies by questionnaires [31,32].

## 2. Materials and Methods

### 2.1. Sample

A sample of Italian midwives willing to participate in the survey were invited through the most frequently used social media, i.e., Facebook and Instagram, to complete a questionnaire. We chose this extremely smart method in order to maximize the final sample size and the willingness to fill a self-administered web survey allowed us to obtain the informed consent to take part in the survey, Thus, participation was voluntary and confidential.

Midwifes were reached through social media, and those willing to fill the questionnaire were enrolled. In this case, they were told that their personal data would not have been recorded in any way; however, we asked them to declare their job activity, and we trusted health care professionals declaring to be midwifes at the time of starting the questionnaire. We could not analyse any other data nor the reason for refusing or agreeing to participate. All cases of incomplete questionnaire were excluded from the analysis.

The survey was built on Google Forms, and sent to Italian midwifes. Data was collected starting from 14 June 2019 to 31 August 2019. The statistical power of the sample to obtain statistically significant results was determined by a freely available on-line web platform. The authors considered the appropriate sample size for an adequate study power considering a confidence level of 99% and a confidence interval of 5% on a total of about 21,000 midwifes working in Italy. The analysis computed a representative sample size of 377 midwifes. The confidence interval also called margin of error is the plus-or-minus figure usually reported in opinion poll results, whilst the confidence level suggests the level of security in excluding wrong answers.

### 2.2. Instruments and Procedures

The questionnaire consisted of a cover page (describing the purpose of the study, as well the methods to ensure anonymity and voluntary participation), and three special sections, including several different items each. In particular, the special sections dealt with:

- demography, actual working schedule, and working experience information;
- the Morningness–Eveningness Questionnaire (MEQ), consisting of 19 questions about personal daily sleep-wake habits and the times of day of preference of certain activities, with assigned points from 0 to 5, giving a possible overall score ranging from 16 to 86. Five categories can be identified: definitely E-type (16–30), moderately E-type (31–41), neither type or I-type (42–58), moderately M-type (59–69), and definitely M-type (70–86). For ease of interpretation, we considered moderately E-type as E-type (16–41 points) and moderately M-type as M-type (59–86 points). Thus, we had three final subgroups: E-types, I-types, and M-types.
- a perception of risk of medication errors survey, based on the “seven rights” (7R) rule of medication administration (right medication, right client, right dose, right time, right route, right reason, and right documentation) [33]. As for risk evaluation, we chose “near misses”, i.e., accidents that do not cause the patient harm [34], since they represent the majority of medication errors [14]. The perception of risk of medication errors was evaluated on the basis of the following questions:
  - Based on the 7R rule, during the last shift, how many times did you (or any of your colleagues) run the risk of making a medication error?
  - Why medication error was about to occur?

### 2.3. Statistical Analysis

For statistical analysis, first a descriptive analysis was performed, including results derived from either MEQ calculated score and personal self-perceived chronotype. The sample was classified according to the self-perception of risk of medication errors, and subgroups by age, working schedule, years of working experience, and chronotype were then compared. Second, a logistic regression analysis was done, considering the self-perception of risk of medication errors as the dependent variable and all the other parameters as the independent ones. Third, a cluster analysis was performed, to determine the phenotype of health care professionals exposed to risk of medication errors based on demography, type of work, and chronotype. IBM Statistical Package for Social Science (SPSS 13.0 for Windows, SPSS Inc., Chicago, IL, USA) was utilized.

## 3. Results

### 3.1. Participants

The final sample included 401 Italian midwives (98.8% women), and the main characteristics are summarized in Table 2.

### 3.2. Chronotype

As for individual chronotype (MEQ score) and age, subgroups were represented as follows: age 23–30 years: 52 ± 8.3; age 31–35 years: 56 ± 8.2; age 36–40 years: 58.2 ± 7.6; age 4–45 years: 58.7 ± 9.2; age 46–50 years: 60.5 ± 8.3; age 51–55 years: 60.4 ± 8; age 56–60 years: 58.5 ± 8.2 (*p* < 0.001).

As for individual chronotype (self-perceived), subgroups were represented as follows: M-type *n* = 115 (28.7%), I-type *n* = 245 (61%), E-type: *n* = 41 (10.2%). Mean MEQ score in groups who perceived M-type, I-type, and E-type was 64.8 ± 5.1, 54.2 ± 5.1, and 44.7 ± 7.1, respectively (*p* < 0.001).

As for individual chronotype (MEQ calculated score), subgroups were represented as follows: definite M-type: *n* = 25 (6.3%); moderately M-type: *n* = 156 (39%); I-type: *n* = 202 (50.3%); moderately E-type: *n* = 16 (4%); definite E-type: *n*= 2 (0.4%).

**Figure 1:**
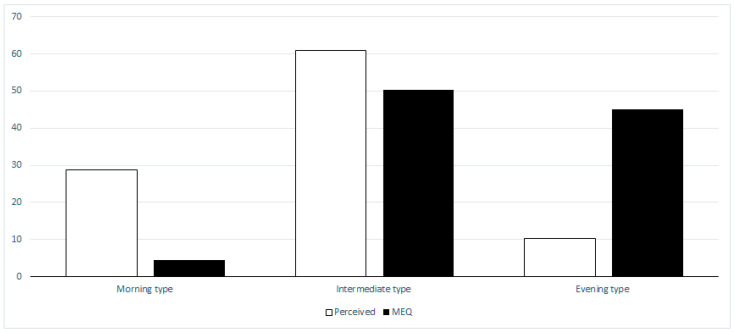
Self-perceived and calculated chronotype distribution of groups. For ease of comparison, the Morningness–Eveningness Questionnaire (MEQ) calculated score was reported considering moderately E-type plus definite E-type as E-type and moderately M-type plus definite M-type as M-type.

Figure 1 reports the distribution of groups by self-perceived and calculated chronotype. For ease of comparison, the MEQ calculated score was reported considering moderately E-type plus definite E-type as E-type and moderately M-type plus definite M-type as M-type.

### 3.3. Perception of Risk of Medication Errors

As for perception of risk of medication errors, subjects with response “no, never” were 208 (51.9%), and subjects with response “yes, at least once” were 193 (48.1%). The MEQ score did not show significant differences between the two groups (56.7 ± 8.4 vs. 56.5 ± 8.9, *p* = NS). No differences between the two groups were found for subgroups by class of age, working shift, years of working experience, and chronotype either.

**Figure 2:**
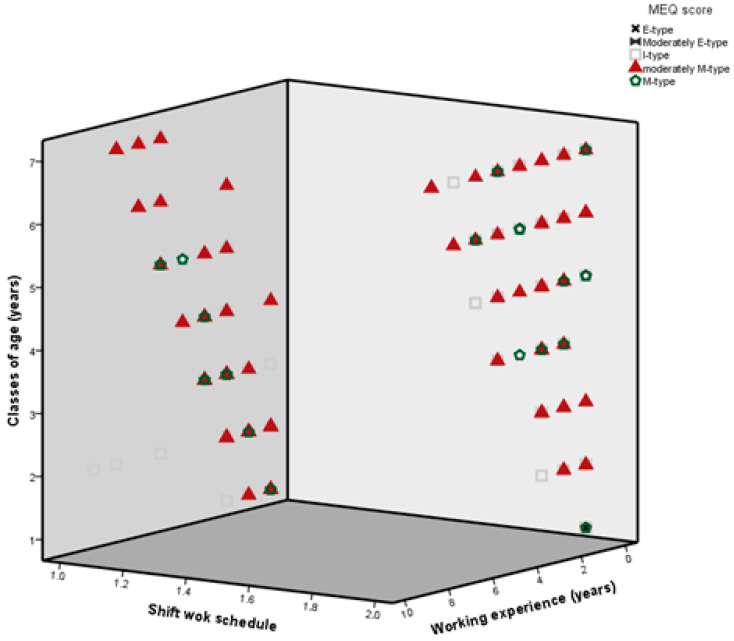
Cluster analysis relating age, shift work schedule, working experience, and MEQ score.

Logistic regression analysis did not show any independent association with perception of risk of medication errors, whereas cluster analysis showed that perception of risk of medication errors was associated with class of age 31–35 years, shift work schedule, working experience 6–10 years, and I-type MEQ score. No perception of risk of medication errors was associated with age 46–50 years, daytime working, working experience 21–25 years, or M-type MEQ score (Figure 2).

## 4. Discussion

The results of this study, addressed to investigate the possible relationship between chronotype and risk of medication errors in midwives, showed that the risk of medication errors was associated with younger age, shift work, relatively low working experience, and being an Intermediate chronotype. This is the first report on the association of shift work and risk medical errors in midwives. A recent multicentre Chinese study showed that sleep quality, social support, job satisfaction, occupational injuries, adverse life events, frequency of irregular meals, and employment type were statistically significant factors influencing fatigue among midwives [35]. We found that younger persons, with reduced working experiences, were more likely to report an increased risk of error. It is possible that experience may help in attenuating the decrease in performance during night shift.

*Errors.* Medication error incidents are more likely to be reported in the morning shift [14,36], but it is common practice that morning therapy is prearranged by the night nursing crew at the end of their shift. Rhythm desynchronization exhibits also gender-specific differences [37], and studies on the circadian and sleep-wake-dependent regulation of cognition in a forced desynchronization protocol showed that accuracy exhibited the largest sex difference in circadian modulation, with the worse performance in women in the early morning hours (at around 6 a.m.) [38]. Even risk taking, a complex form of decision-making that involves calculated assessments of potential costs and rewards, may play a role in the determination of errors. Both gender-specific and chronotype differences exist, since males report higher propensity for risk-taking, in particular E-types. However, although there is no significant difference in risk propensity or risk-taking behaviour across chronotypes in males, E-type females significantly report and take more risk than other chronotypes [39].

*Chronotype.* Chronotype is also strongly implicated in performance tasks, also making reference to the so-called “synchrony effects”, i.e., superior performance at optimal and inferior performance at suboptimal times of day. A study aimed at evaluating the effect of individual differences in chronotype on performance task, evaluated a sample of M-type and E-type women during a driving session in morning (8 a.m.) and evening (8 p.m.). A vigilance decrement was found when E-type participants drove at their nonoptimal time of day (morning session). In contrast, driving performance in the M-type group remained stable over time on task and was not affected by time of day [40]. By contrast, studies on subjects tested for mean reaction times, error rates, and efficiency of three attentional networks (alerting, orienting, and executive control/conflict) at two time points (time 1 or baseline at 8 a.m.; time 2, after 18-h sustained wakefulness at 2 a.m.), showed that E-types participants outperformed M-types on incongruent time, i.e., deep night [21]. On one hand, negative effects of sleep impairments seem to be confirmed to affect more E-types than other chronotypes. An Australian online survey study conducted on paramedics (age 39 ± 12 years; 54% women; 85% rotating shift-workers; 57% I-types, 32% M-types, 11% E-types), showed significantly higher depression scores, anxiety, poorer sleep quality, and reduced general well-being in the E-types, compared with M-types [41]. Our study identifies Intermediate-type as the group at higher risk of error. On one hand, this result is contradictory with respect to the available literature. On the other, this result, if confirmed, is extremely interesting since Intermediate chronotype represents the most frequent circadian preference and not only in our sample. Thus, the finding that younger midwives, with relatively limited working experience, and even with chronotype extremes, are exposed to higher risk of error during their night shift, raises serious concern.

*Limitations.* We are aware of several limitations to this study: (a) cross-sectional design, based on data collected through a web survey, therefore the sample could be sized only by social media users only; (b) the MEQ score, even if used in the majority of studies for assessing morningness–eveningness preference, does not categorized for different ages [42]; (c) to evaluate the risk of medication error, we considered the general definition of near misses. Although it is largely the most frequent incident reported, we did not differentiate between near misses and no harm incidents [43]; (d) individual perception of the risk of medication errors is not a validated item yet; (e) logistic regression analysis did not identify independent factors; (f) we could not evaluate any gender effect, as most of the sample were women, as usually occurs for studies on nurses and midwives. However, there are also some positive aspects: (a) the web survey method warranted speed, and even in a short timeframe all different classes of age were represented; (b) we found that perception of chronotype was different from the effective profile identified by a well-validated score. This concept could help for future studies and also for practical applications since when workers (health care personnel in particular) ask for health measures, they very often make reference to personal perception.

*Coping strategies*. Midwifery and nursing are acknowledged as stressful occupations, and the negative impact of high stress levels often requires coping strategies. Of these, the eleven most used have been identified via interview material: drinking alcohol, smoking, using the staff social club, using social networking websites, exercising, family activities, home-based activities, outdoor activities, avoiding people, displacement, and sleep [44]. Unfortunately, it is evident that some of these coping strategies are unhealthy and extremely concerning. Moreover, due to the stress burden, absenteeism is becoming a significant global problem, and taking a “mental health day” as sickness absence is a common phenomenon, taken by more than one half of nurses and midwives, according to an online cross-sectional survey in Australia [45].

## 5. Conclusions

With this study, we found that younger midwives, with lower working experience, engaged in shift work and belonging to an Intermediate chronotype, seem to be at higher risk of potential medication error. At least to the best of our knowledge, this is the first web survey study addressed to the relationship between chronotype, shift work, and perceived risk of medication error in midwives. Since morning hours seem to represent highest risk frame for female healthcare workers and shift work is not always aligned with individual circadian preference, our preliminary reports could stimulate further specific research aimed to practical applications. For example, assessment of individual chronotype and sleep attitude in healthcare personnel, by means of validated questionnaires, just previously suggested in terms of prevention of metabolic diseases [46], could provide easy and inexpensive method to identify subjects at potential higher risk of circadian disruption. Again, possible countermeasures, such as time-scheduled naps during night-shifts, have been positively tested on female nurses, who showed significantly greater increments in performance between 3:00 and 7:00 a.m. on nap versus no-nap nights [47]. Last but not least, attention to shift work consequences, together with specific training programs, could help to reduce medication errors and improve patients’ safety [48].

## Figures and Tables

**Table 1:** Association between chronotype and main health issues [Fabbian 2016] (Symbols: ↑ Increased; ↓ Decreased).

Main Health Issues	Chronotype	Main Findings
General and cardiovascular health	Evening-type	Unhealthy diet ↓ Physical activity ↑ Smoking Metabolic syndrome Diabetes mellitus
Psychological & psychopathological issues	Evening-type	↑ Common mental disorders ↑ Depression symptoms ↑ Anxiety symptoms Nightmares Risk-taking behaviour
	Morning-type	↑ Health-related quality of life
Sleep & sleep-related issues	Evening-type	Later bedtime and wake-up ↓ Sleep duration ↓ Sleep quality ↓ Sleep quantity ↓ Sleep efficiency

**Table 2:** Characteristics of the whole population of midwifes.

Number of Participants (*n*)	401
Female (*n* (%))	396 (98.8)
Mean age (years)	38.5 ± 10.1
age 23–30 years (*n* (%))	102 (25.5)
age 31–35 years (*n* (%))	81 (20.0)
age 36–40 years (*n* (%))	79 (19.8)
age 41–45 years (*n* (%))	37 (9.2)
age 46–50 years (*n* (%))	37 (9.2)
age 51–55 years (*n* (%))	30 (7.5)
age 56–60 years (*n* (%))	35 (8.8)
University degree (*n* (%))	32 (8)
High school degree (*n* (%))	369 (92)
Shift work schedule (*n* (%))	293 (73)
Mean working experience (years)	11.7 ± 8.9
